# Modelling and optimization of operating parameters of an electronic cell type metering mechanism for urea super granules (USG) using EDEM-RSM approach

**DOI:** 10.1038/s41598-026-43407-w

**Published:** 2026-04-02

**Authors:** Sidhartha Sekhar Swain, Tapan Kumar Khura, Prabhakaran Arjun, Hari Lal Kushwaha, Roaf Ahmad Parray, Deepak Saini, Manojit Chowdhury, Pramod Kumar Sahoo, Pankaj Malkani, Kirttiranjan Baral, Nadhir Al-Ansari, Mohamed A. Mattar, Ali Salem

**Affiliations:** 1 Division of Agricultural Engineering, ICAR-Indian Agricultural Research Institute, New Delhi, 110012 India; 2 School of Water Resources, IIT, Kharagpur, West Bengal 721302 India; 3 Department of Agriculture and Food Engineering, IIT, Kharagpur, West Bengal 721302 India; 4 Krishi Vigyan Kendra, Narkatiaganj, Dr. Rajendra Prasad Univeristy, PUSA, Samastipur, Bihar India; 5 ICAR-National Academy of Agricultural Research Management, Hyderabad, 500030 India; 6 Department of Civil, Environmental and Natural Resources Engineering, Lulea University of Technology, Lulea, 97187 Sweden; 7 Prince Sultan Bin Abdulaziz International Prize for Water Chair, Prince Sultan Institute for Environmental, Water and Desert Research, King Saud University, P.O. Box 2454, Riyadh 11451, Saudi Arabia; 8 Civil Engineering Department, Faculty of Engineering, Minia University, Minia, 61111 Egypt; 9 Structural Diagnostics and Analysis Research Group, Faculty of Engineering and Information Technology, University of Pécs, Pécs, 7622 Hungary

**Keywords:** EDEM, RSM, USG, Simulation, Optimization, Agroecology, Electrical and electronic engineering

## Abstract

The development of an efficient electronic metering system is crucial for the successful deployment of urea super granules (USGs) in agricultural fields. Key parameters influencing the performance of the cell-type metering system for USG application include cell area, peripheral speed of the cell, and the level of hopper filling. Simulation of the metering system operation was conducted using EDEM, with a focus on optimizing input parameters to achieve 100% cell fill, maximum qualified rate for single cells, and minimization of missing or multiple cells. An electronic metering system was implemented in a soil bin, employing a Nema 23 stepper motor to control the peripheral speed of the cell. The synchronization with the transplanter was ensured through a rotary encoder, and various cell sizes were achieved using 3D printing. Using the EDEM-RSM approach, the optimal operating conditions based on the soil bin study were found as cell area of 1088 mm^2^, a metering roller peripheral speed of 0.24 m/s, and a 75% hopper fill. Under these conditions, the system demonstrated a 97% cell fill, a 91% qualified rate for single cells, with only 3.2% missing cells and 4.5% multiple cells. Quality class of metering was found as good based on indices such as missing index, multiple index, and quality of feed index, which were found as 3.2%, 4.5%, and 92.3%, respectively. The study demonstrated the robust predictive capability of the EDEM-RSM model in evaluating the performance of the cell-type metering system. These findings offer valuable insights for researchers and manufacturers seeking to optimize the development of automated ultrasonic (USG) applicators.

## Introduction

Atmospheric methane (CH4) and nitrous oxide (N2O) are significant greenhouse gases (GHGs) with global warming potentials (GWPs) approximately 25 times and 298 times higher than that of carbon dioxide (CO2) over a 100-year time span^1^. Excessive utilization of nitrogen fertilizers leads to heightened emissions of greenhouse gases from agricultural activities, thereby contributing to an elevated Carbon Footprint (CF)^2^. The practise of N deep placement, which has been shown to be a successful strategy to improve N use efficiency and reduce N loss, has been suggested by researchers as a solution to such issues^3^. Researchers in Bangladesh have discovered that placing urea super granules (USG) deep in the middle of four hills increases nitrogen utilisation efficiency (NUE) from 35% in case of PU to 63 to 67% in dee placement and also increases yield by 30 to 40% when cmpared to broadcasted urea^4,5^. Although the manual method of placing urea deeply is effective, farmers are frequently deterred from using it because of the significant labour costs and hard labour involved^6^. Therefore, using an applicator for basal application could be a simple, labour-saving, accurate (i.e., human error-free), and highly efficient technique of placing urea briquettes. With the advancement of technology in the field of agriculture, several initiatives have been undertaken in the past to develop both continuous and non-continuous operation types of USG applicators^7–9^. The efficient operation of USG applicator is highly dependent upon the efficient metering of USGs. Several studies have been reported that using electronic system instead of mechanical system improved the uniformity of application in planting^10–13^. The different studies on use of advance metering systems like electronic metering system in groundnut^14^, electronic cup chain type metering device in potato^15^, electro-mechanical system in corn^16^ and electro-mechanical system for sowing of pregerminated paddy seeds^17^ have showed efficient results in terms of precision seed spacing, miss index, multiple index and precision index. The extension of such approach to fertilizer metering needs to be critically analysed.

A comprehensive investigation is required, particularly to understand the relationships between machine parameters and performance in the context of fertilizer metering. The complexity of the fertilizer metering process—characterized by the movement of fertilizer particles and their interactions with the machine—poses a significant challenge in conducting experiments to establish these relationships^18^. A more effective approach is to employ computer simulation, which has the capability to handle various types of particles within a system and accurately capture their motion at any moment. The discrete element method (DEM) has been widely recognized as a valuable simulation tool for effectively simulating discrete particles in agricultural applications. Discrete Element Method (DEM) simulations, which begin with the generation of USG particles and proceed through model calibration and validation, have gained traction in recent research. Scientists have increasingly turned to DEM to explore the interactions between granular materials and the operational components of agricultural machinery. This method has proven highly effective in minimizing both the time and costs typically associated with the design and development of agricultural equipment. The complex nature of granular flow, such as that involved in fertilizer or seed metering, makes DEM particularly valuable for studying these systems. By simulating the behavior of particles within machinery, DEM enables researchers to optimize performance without extensive physical prototyping. Currently, there is a notable shift toward utilizing DEM for seed-metering mechanism research, reflecting its growing importance as a tool for advancing the precision and efficiency of agricultural machinery design. Shi et al.^19^, employed the DEM approach to examine the impact of distinct seed-metering plates on the performance of pneumatic combined-hole precision seed-metering devices. Li et al.^20^, employed the discrete element method to analyse an internal-seeding device and wheat seeds and observed a good agreement between simulation and experimental result. To enhance the seed metering device performance using air-suction rollers, Zhang Kun^21^, employed discrete element analysis to conduct numerical simulations varying the seed layer height, vibration frequency, and vibration angle. Corresponding metrics including qualified rate, leak seeding rate, and re-seeding rate serving as evaluation indices. Experimental outcomes were validated the alignment between observed and optimal results. To enhance seed-metering precision, Shi et al.^22^, performed DEM simulations on a horizontal circular-plate precision seed meter. Additionally, DEM has been effectively employed in analyzing particle dispersion mechanisms in diverse agricultural machinery contexts^23–28^.

Type of metring system used for USG metering is cell type^29^. In cell type metering system, the cells are designed to be filled with a precise number of USGs and emptied into a tube with each rotation^30^. Each cell of the metering roller should ideally hold one USG and meter it into the tube (i.e., 100% cell fill), which is necessary to achieve the uniform distribution^31,32^. Therefore, this research was undertaken to find the optimal operating settings under laboratory circumstances before moving to the field for a sizable reduction in the non-uniformity of USGs distribution. The size of the cell on the metering roller for USG singulation, the peripheral speed of the roller and level of hopper filling all need to be optimised in order to get correct spacing of USGs. Present study was carried out at the Division of Agricultural Engineering, IARI New Delhi, India.

To clearly position the contribution of this study within the existing body of knowledge, Table 1 summarizes the main characteristics of previously reported metering mechanisms and highlights how the present work advances the state of the art. Unlike prior studies that primarily relied on either mechanical prototypes or DEM simulations for seed metering, the present research integrates DEM modelling, response surface optimization, and laboratory validation specifically for USG fertilizer, which has distinct physical and flow properties compared with seeds. This combination provides a more robust and quantitative framework for predicting and optimizing metering performance. The incremental advances achieved in this work include the development of an electronic cell-type metering mechanism, optimization of operating parameters using RSM, and successful validation through soil-bin experiments, collectively contributing to the design of continuous-operation USG applicators.

**Table 1:** Comparison of the proposed USG metering system with related studies in the literature.

Study	Metering mechanism	Simulation/modelling used	Parameters optimized/evaluated	Key performance results	Limitations in literature	Incremental advance in the present study
Shi et al. (2015)	Pneumatic combined-hole maize metering plate	DEM simulation	Seed filling performance	Good agreement between simulation and lab tests	Focused on seeds; not suitable for heavy fertilizer briquettes; no optimization framework	Our study extends DEM modelling to USG fertilizer, which has different physical behaviour.
Li et al. (2011)	Internal-filling device	DEM analysis	Seed motion and filling	Verified simulation–experiment match	Did not optimize continuous-operation fertilizer metering	First study to model cell-type USG metering using DEM.
Zhang Kun (2017)	Air-suction roller	DEM + experimental optimization	Seed layer height, vibration parameters	Improved seed singulation	Pneumatic mechanism unsuitable for dense USGs; no multi-factor optimization	Introduces mechanical cell-type metering suitable for USGs with systematic RSM optimization.
Shi et al. (2014)	Horizontal disc metering	DEM simulation	Seed singulation	Provided qualitative insights	Performance metrics limited to small seeds	First to quantify cell fill, missing %, multiple % specifically for granular fertilizer.
Gao et al. (2021)	High-speed seed metering	DEM analysis	Particle motion	Improved high-speed accuracy	Focus on high-speed planting, not fertilizer	Our system addresses low-speed, high-mass particle metering relevant to USG application.
Hossen et al. (2013), Wohab et al. (2017)	Manual USG applicators	No modelling; experimental only	Manual placement mechanisms	High labour requirement; inconsistent placement	No automation or optimization	Present work delivers fully electronic metering eliminating labour dependence.
Present study (2025)	Electronic cell-type USG metering system	Integrated DEM simulation + RSM optimization + laboratory validation	Cell area, peripheral speed, hopper fill	97% cell fill, 91% single-cell qualified rate, 3.2% missing, 4.5% multiple	–	Novel integrated framework: DEM + RSM; first optimization of USG metering parameters; validated in soil bin; applicable for automated USG applicators.

In this study, modelling of USG metering system was done by discrete element method using EDEM software. The operating and machine parameters of metering system like cell area, peripheral speed of roller and level of hopper filling were optimized to find the 100% cell fill, maximum qualified rate of single cell and minimum missing & multiple cell percent using Response surface method. Additionally, the optimized parameters were validated by conducting the experiment in soil bin. The output of this study may be helpful for the farmers as well as the manufacturers to fabricate continuous operation type USG applicator.

## Materials and methods

### Experimental setup for evaluating USG metering system

**Fig. 1:**
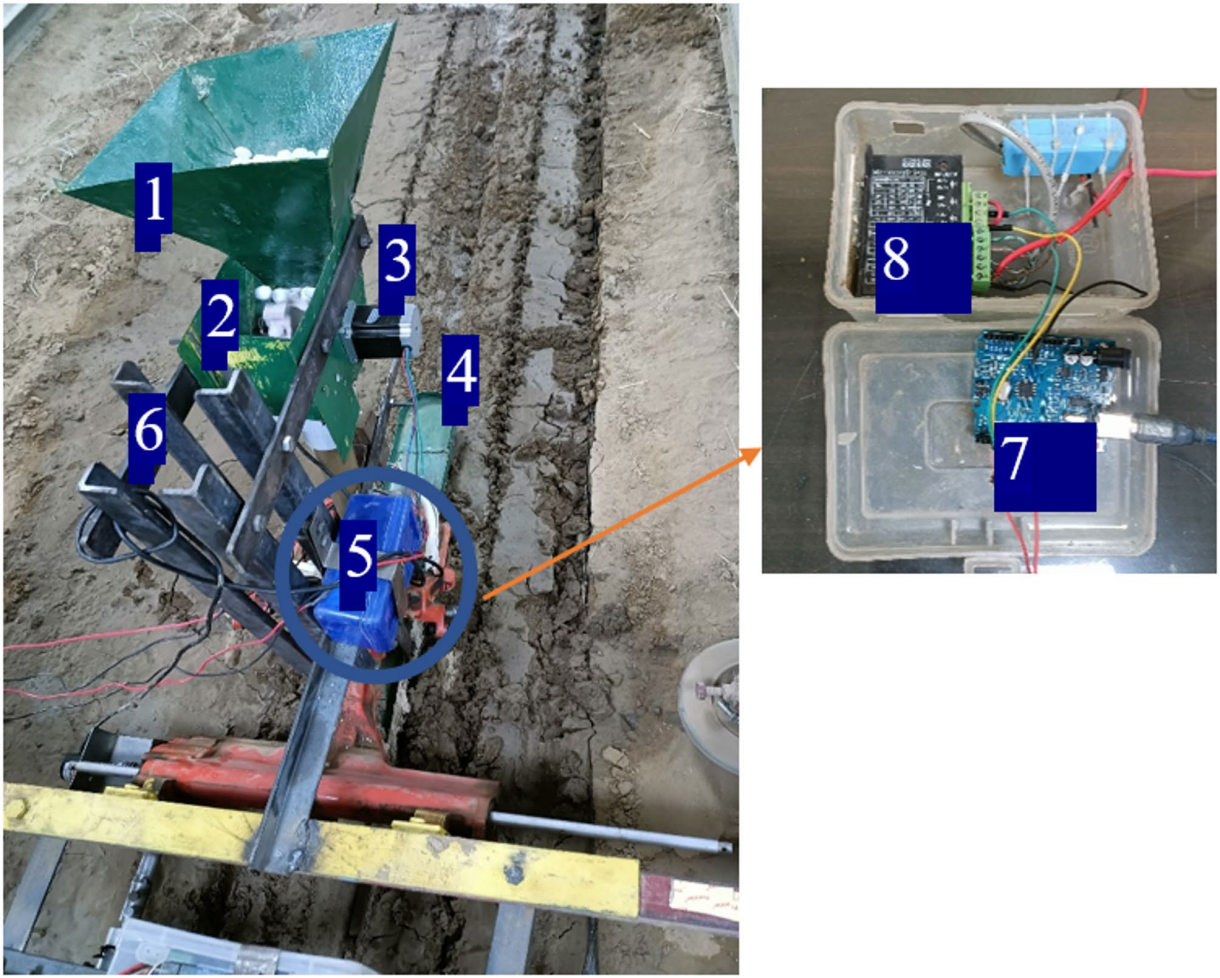
Laboratory setup for the experiment. 1—Hopper, 2—Seed metering system, 3—Nema 23 stepper motor, 4—Float, 5—Electronic system, 6—Frame, 7—Arduino Uno, 8—TB 6600 motor driver.

**Fig. 2:**
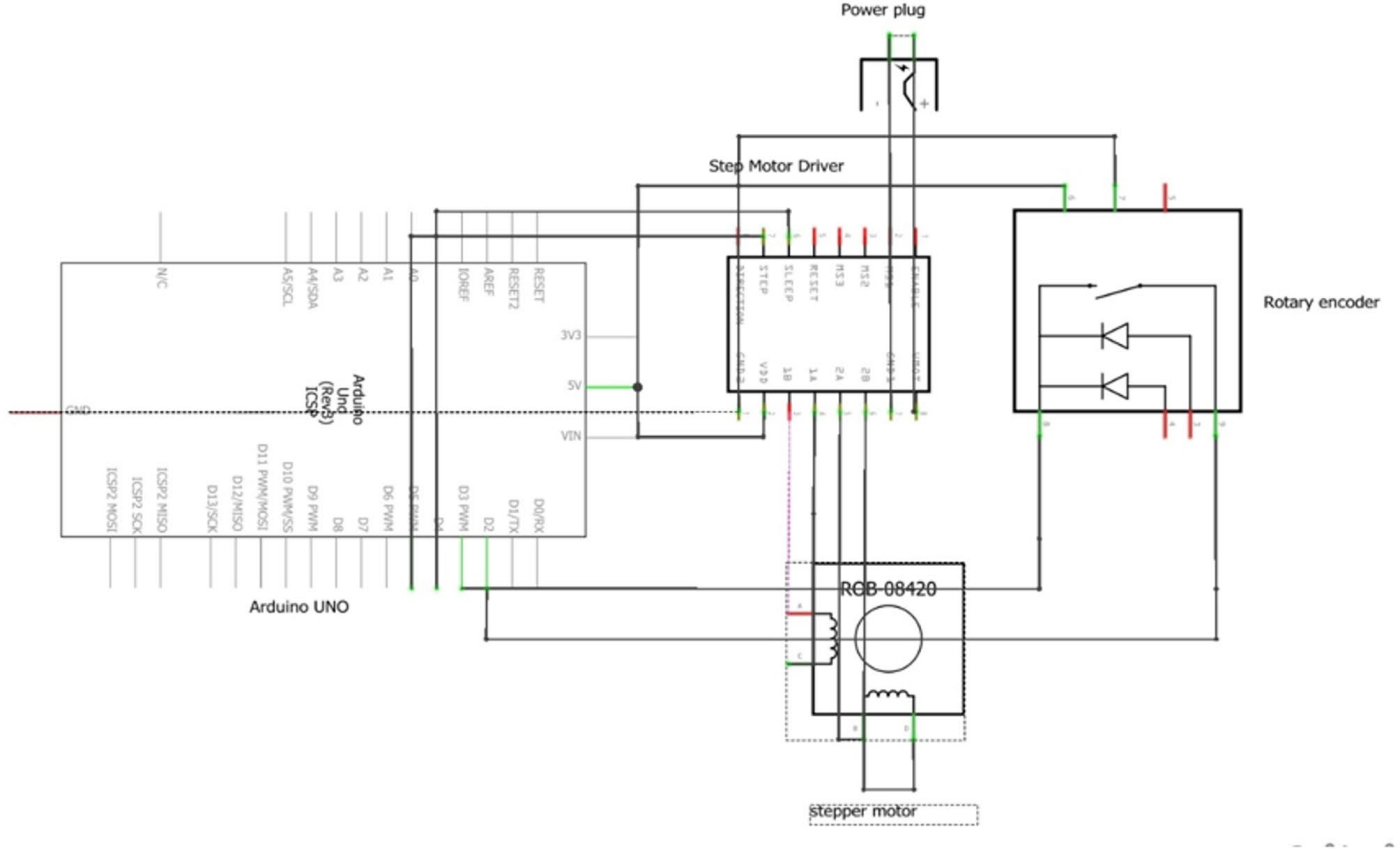
Circuit diagram for the operation of metering system.

**Fig. 3:**
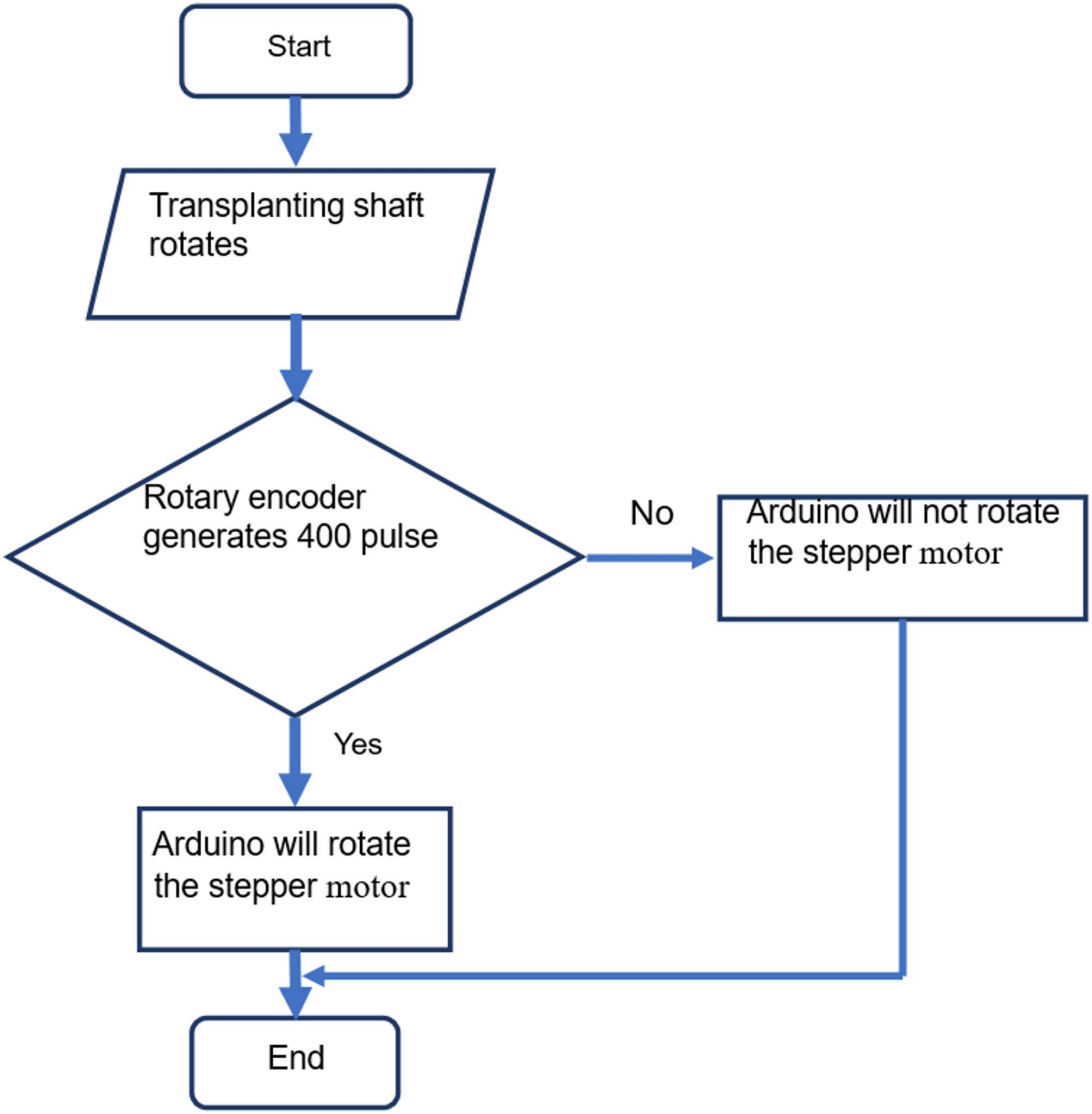
Flow chart of the operation of metering system.

The test setup was developed to evaluate the metering unit with varying parameters in the soil bin. The lab set up consisted of 2 row transplanter model, fertiliser box, metering rollers, Arduino Uno, 12 V battery, Nema 23 stepper motor and TB 6600 stepper motor driver. Transmission system of a 2-row paddy transplanter along with transplanting fingers was mounted on the frame of the soil bin for this study. The metering roller consisted of 4 cells on its periphery. The cell area was taken as per the engineering properties of USG (Table 2). The metering roller was coupled to a stepper motor via a shaft and a bush. The speed of the metering roller was synchronized with the speed of the transplanting shaft via a rotary encoder. A rotary encoder (400 PPR) was fitted on the transplanting shaft for sensing its revolution. Transplanting finger actuated once (transplant 1 hill) per revolution of transplanting shaft. As per the recommendations, an USG was to be placed at the centre of the four hills^33,34^. Therefore, the metering roller rotated 0.25 revolution per revolution of transplanting shaft. Each revolution of transplanting shaft generates 400 pulses by the rotary encoder. Using TB 6600 stepper motor driver 1600 pulse per revolution was selected for the operation of stepper motor using Arduino Uno micro controller board. The laboratory system, the circuit diagram and flow chart of electronic metering system has been presented in Figs. 1, 2 and 3, respectively.

**Table 2:** Engineering properties of USG.

Physical properties	Mean	Standard error
Length (mm)	16.60	0.63
Breadth (mm)	15.30	0.45
Thickness (mm)	7.2	0.61
Angle of repose	33.39	1.49
Bulk density (g/cm3)	0.75	0.01
Particle density (g/cm3)	1.26	0.02

### Determination of operating parameters of the USG metering system

In this study, cell fill, qualified rate of single cell, missing cell percent, and multiple cell percent were determined under different combinations of input parameters, each conducted in three replications. As the shape of USG is ellipsoidal, the surface area of a single particle was calculated using Knud Thomsen’s formula and found to be 1060 mm². Based on this value, the metering roller, consisting of four cells on its periphery, was designed by selecting a cell area ranging from 75% to 125% of the mean USG surface area. This proportional range was chosen to ensure adequate singulation while minimizing the likelihood of multiple pickup, as cell dimensions in precision metering systems are generally recommended to closely correspond to particle size for balanced filling and discharge performance.

The operating speed of the walk-behind paddy transplanter under muddy field conditions was observed to vary between 2 and 3 km h⁻¹^35^. Considering a mean plant spacing of 20 cm and four cells on the metering roller periphery, the corresponding peripheral speeds of the roller were calculated as 0.22 m s⁻¹ and 0.34 m s⁻¹ for the minimum and maximum forward speeds, respectively. These limits represent realistic field operating conditions and account for the mechanical synchronization between the transplanting shaft and the metering roller. Previous studies on granular and seed metering devices have indicated that lower peripheral speeds may increase the probability of multiple pickup, whereas higher speeds reduce filling time and may increase missing rates. The hopper filling level was varied between 25% and 75%. The lower limit ensured sufficient particle availability at the cell entrance, while the upper limit prevented excessive overburden pressure that could adversely affect particle flow characteristics. Similar hopper fill ranges have been reported in earlier investigations of seed and granular metering mechanisms.

Accordingly, the upper and lower limits of the independent variables presented in Table 3 were defined based on material properties of USG and practical operational constraints of the developed metering system. These limits were therefore considered appropriate for developing a second-order response surface model.

**Table 3:** Research plan laboratory testing.

Sl no.	Parameters	Maximum value	Minimum value
	**Independent parameters**		
1	Cell Area (mm^2^)	1325	795
2	Peripheral Speed (m/s)	0.34	0.22
3	Level of hopper filling (%)	75	25
	**Dependent parameters**		
1	Cell fill (%)		
2	Qualified rate of single cell (%)		
3	Missing cell percent (%)		
4	Multiple cell percent (%)		

### Plan for laboratory studies

Objective of this study was to find the optimum set of machine and operating parameter to get desired output of developed metering mechanism. Parameters being measured were cell fill, Qualified rate of single cell, missing cell percent and multiple cell percent (Table 4).

**Table 4:** Parameters evaluated in the laboratory testing.

Parameters	Formula	Remark
Cell fill (F_c_,%)	F_c_= $\frac{Ns\times100}{Nc}$	Ns = Total number of metered USGs. Nc = Total number of cells passing through the discharge point [39].
Qualified rate of single cell (Q_s_, %)	Q_s_ = $\frac{N1}{N}$ × 100	N_1_ = Number of cells carrying single USG N = Total number of cells during a given time
Missing cell percent (C_miss_, %)	C_miss_ = $\frac{L1}{N}$ **×**100	L_1_ = Number of spacing greater than 1.5 times theoretical spacing. N = Total number of observations. (ISO 7256/1–1984(E))
Multiple cell percent (C_mult_, %)	C_mult_ = $\frac{L2}{N}$ **×**100	L_2_ = Number of spacing lesser or equal to 0.5 times theoretical spacing. (ISO 7256/1–1984(E)).

### Experimental procedure for laboratory study

As per the CCD design of RSM the operating parameters of the developed system consisted of 30 set of conditions (Table 7). Simulation for 30 sets of conditions was done by using discrete element method.

#### Particle model of single USG in EDEM

**Fig. 4:**
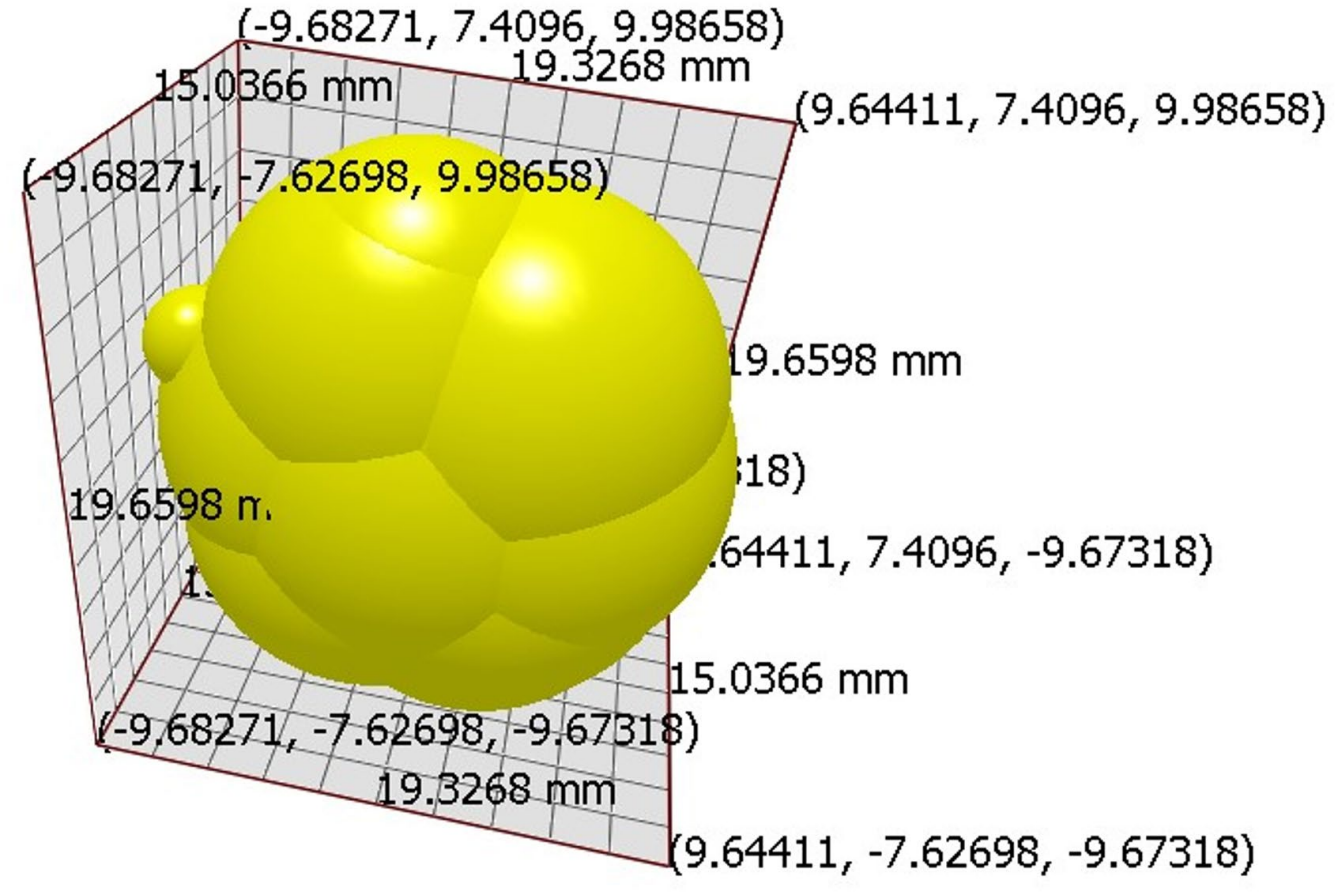
Model diagram of USG in EDEM.

Particle modeling in EDEM, utilizing the EDEM, serves as a fundamental and powerful tool in understanding and simulating the behaviour of individual particles within bulk materials. This approach allows for the detailed analysis of granular interactions, capturing the dynamics of particle motion, collisions, and contact forces in a realistic manner. The particle model of a single USG was established by filling the shape of USG with the spheres. Total 11 spheres were used to create the model of USG (Fig. 4). Properties of the USG created has been presented in Table 5.

**Table 5:** Properties of model USG in EDEM.

Parameters	Value
Mass (kg)	0.0016687
Volume (m^3^)	3.034 × 10^− 6^
Moment of inertia X (kg m^2^)	5.1849 × 10^− 8^
Moment of inertia Y (kg m^2^)	6.4185 × 10^− 8^
Moment of inertia Z (kg m^2^)	5.0430 × 10^− 8^

#### Geometric modelling of USG metering system for EDEM simulation

**Fig. 5:**
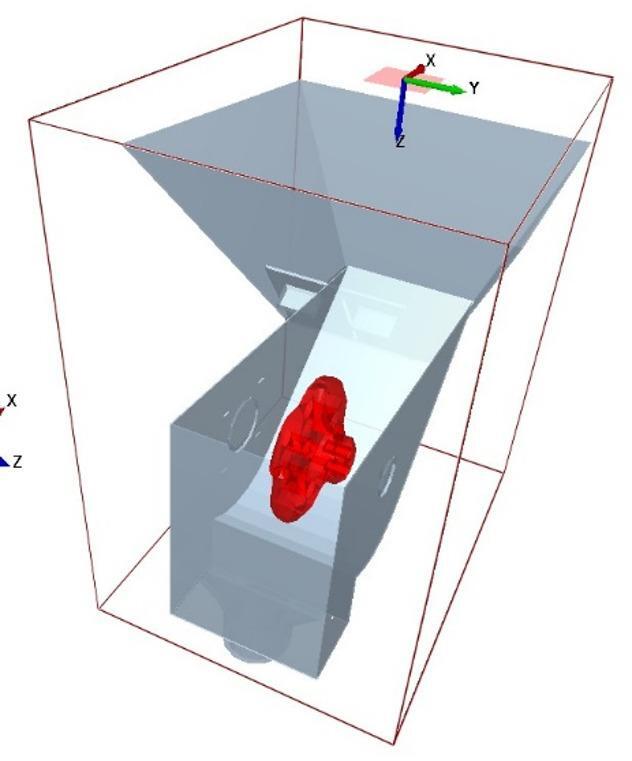
Model diagram of seed metering system in EDEM.

**Fig. 6:**
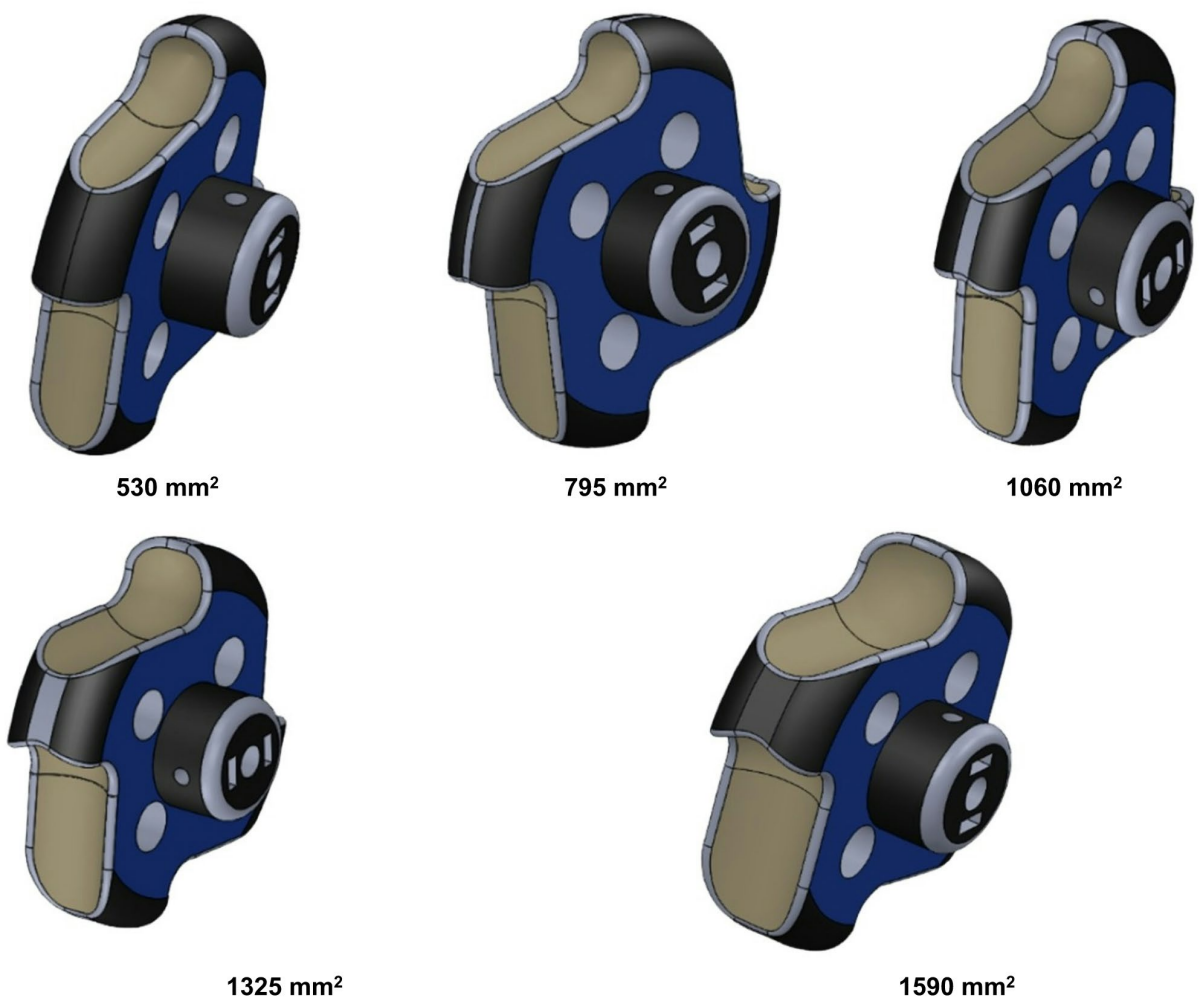
Design diagram of metering roller with different cell area.

Geometric modeling in EDEM is a critical aspect that involves accurately representing the physical characteristics and shapes of particles within a simulated environment. This process is essential for capturing the intricacies of particle interactions and their spatial arrangements in bulk material simulations. Through geometric modeling, EDEM enables the simulation of various particle shapes, sizes, and properties, providing a realistic representation of granular materials in diverse industrial applications. USG metering system involves hopper, metering roller, stepper motor, shaft for holding metering roller, and bearing. Three-dimensional model of the components was designed using solidworks and imported into the EDEM software. Material of the parts were allocated according to their material of construction. A virtual particle factory of size 40 × 40 mm was created above the primary hopper which creates 500 particles for this study (Fig. 5). Particle factory generates particle randomly, which falls on the primary hopper and eventually flows into the auxiliary hopper and the metering roller. linear rotational motion was given to the metering roller which rotates around the shaft holding it. Five numbers of metering rollers with cell area of 530, 795, 1060, 1325 and 1590 mm^2^ as per the requirement in Table 7 were designed using the CAD software solidworks (Fig. 6).

#### Contact model and parameter setting in EDEM environment

The Hertz-Mindlin (no-slip) contact model was utilized in EDEM to accurately and efficiently simulate the interaction between particles, including both particle-particle interactions and particle-boundary interactions (such as the metering roller and USG). Proper parameter settings for these global variables are essential for achieving precise and reliable simulation results in EDEM^36^. Urea has a poison’s ratio of 0.3 and shear modulus of 0.3 × ${10}^{9}$ Pa^37,38^. The metering mechanism was 3D printed, so the poison’s ratio and shear modulus of PLA material and has a poisons ratio and shear modulus of 0.3 and 1.5–4 × ${10}^{9}$ Pa^39^ (Table 6).

**Table 6:** Setting of the material physical and mechanical contact property parameters.

Material	Parameter	Value
USG	Poisson’s ratio	0.3
	Shear modulus (Pa)	0.3 × ${10}^{9}$
	Density (kg/m^3^)	750
PLA material	Poisson’s ratio	0.3
	Shear modulus (Pa)	4 × ${10}^{9}$ Pa
	Density (kg/m^3^)	1240
USG – USG	Coefficient of restitution	0.1
	Static friction coefficient	0.2
	Rolling friction coefficient	0.05
USG – PLA	Coefficient of restitution	0.31
	Statis friction coefficient	0.6
	Rolling friction coefficient	0.1

By employing the CAD design of the metering roller, the cell area was adjusted in EDEM environment. Similarly, level of hopper filling was manipulated by generating an appropriate number of particles.

#### EDEM model calibration for fertilizer metering simulation

**Fig. 7:**
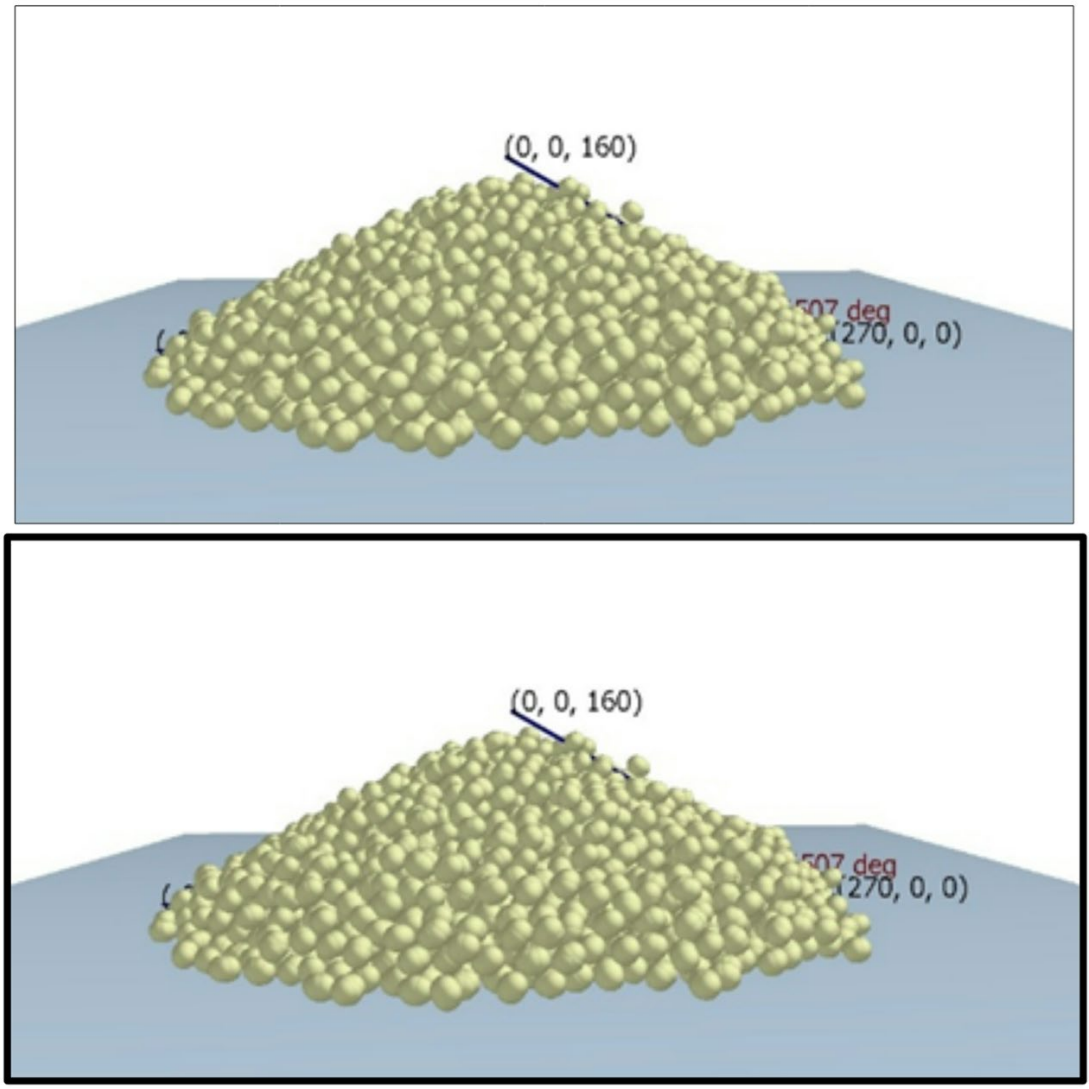
Generation of USG pile for angle of repose measurement.

In the physical experiment, a hollow cylindrical tube with an internal diameter of 100 mm and a height of 150 mm was placed vertically on a flat mild steel base plate. The cylinder was gently filled with USG particles without applying external compaction to preserve their natural packing condition. After filling, the cylinder was lifted vertically at a controlled speed of 50 mm s⁻¹, allowing the particles to form a stable conical pile under gravitational action, as illustrated in Fig. 7. The selected lifting speed ensured quasi-static conditions during pile formation and minimized dynamic particle scattering, which could otherwise influence the measured angle. The experiment was conducted in three replications, and the average value was considered for calibration. The same geometric dimensions and lifting conditions were reproduced in the EDEM environment to maintain consistency between the physical and numerical tests. The simulated angle of repose was determined using the measurement tools available within the software. The experimentally measured angles were 32.04°, 31.97°, and 33.94°, resulting in an average of 32.65°. The corresponding simulated value was 30.50°, yielding a relative error of 6.58%. This deviation falls within the acceptable range typically reported for DEM calibration of agricultural granular materials. Accordingly, the selected material and contact parameters were considered appropriate for subsequent simulation of the metering process.

#### Sensor allocation in EDEM simulation

**Fig. 8:**
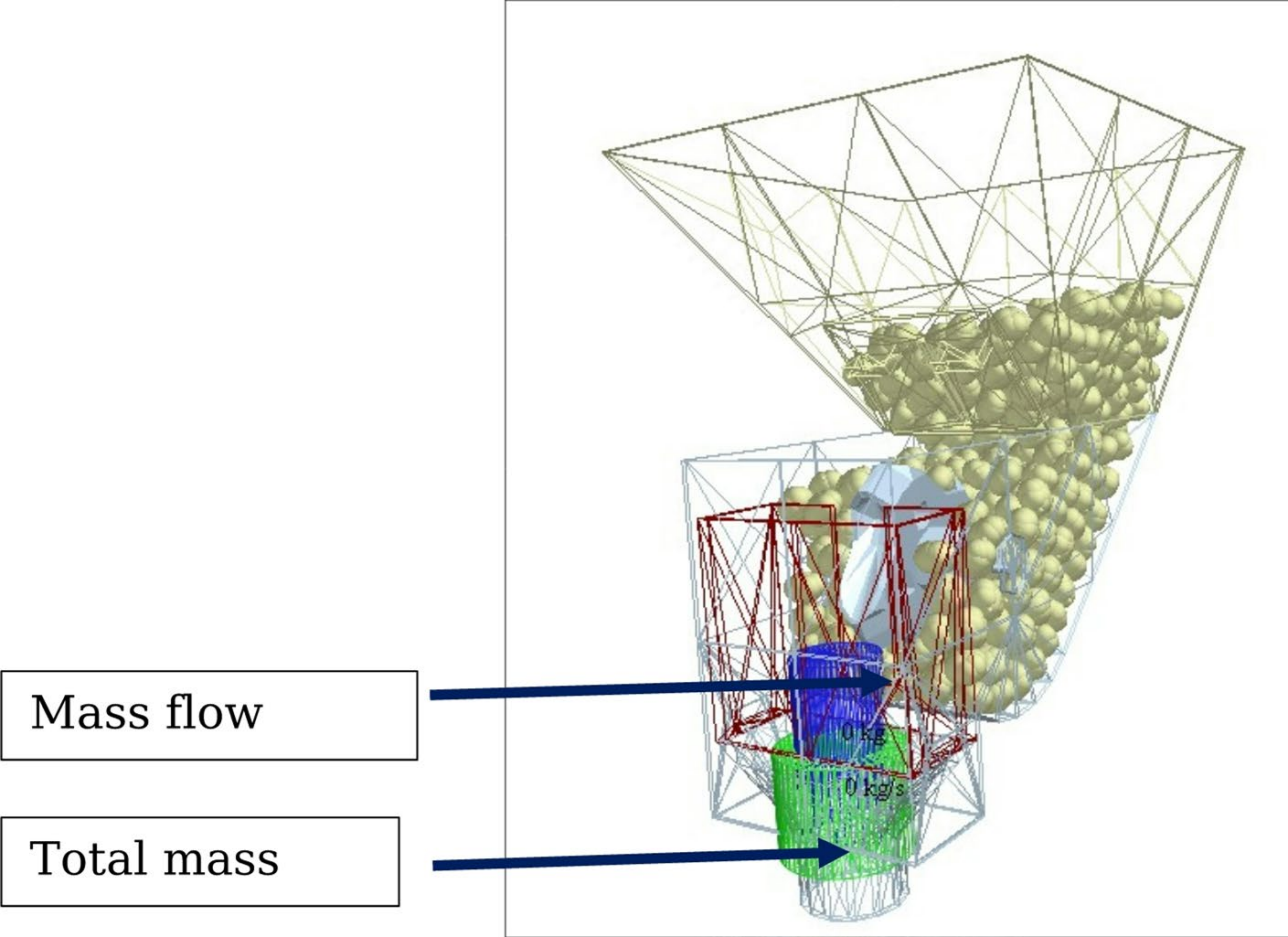
Sensor allocation to metering system.

In EDEM simulations, various sensors are employed to gather data and monitor different aspects of the simulated system. These sensors play a crucial role in capturing relevant information during the simulation and can be categorized into several types based on their specific functionalities. Sensors used in this study involved number sensors and mass sensors (both mass flow rate sensors and total mass sensors). The allocation of sensors to the metering system has been shown in Fig. 8.

### Cost economics of the developed system

The bill of materials for the USG applicator was compiled, and an estimated cost of fabrication was calculated. To determine the total cost of the USG applicator, both fixed and variable costs were taken into account. The evaluation of cost economics for the USG applicator considered the following cost components.

#### Total cost of production

Total cost of operation was determined as the sum of fixed and variable cost.

A) Fixed cost.

1. Depreciation.
2. Interest.
3. Insurance and taxes.
4. Shelter.

B) Variable cost.

1) Repair and maintenance.

The total hourly cost of operating the machine was computed, taking into account both fixed and variable costs. Similarly, the cost of operating the transplanter was calculated using the same procedure. The variable cost included expenses for fuel, lubrication, and the operator’s wages. To determine the overall cost of operating the USG applicator, the hourly cost of operation for both the applicator and the transplanter were added together and expressed on an hourly basis. This hourly cost was then converted into an area basis by multiplying it with the effective field capacity of the machine. Additionally, the Break-Even Point (BEP) and Pay Back Period (PBP) were calculated to assess the financial viability of the prototype USG applicator.

#### Break-even point

The break-even point represents the equilibrium where gains match losses. It signifies the moment when an investment begins to yield a positive return. At the break-even point, there is neither profit nor loss; it marks the threshold of profitability when prices and margins are set.

$$\mathrm{B}\mathrm{E}\mathrm{P}=\frac{\mathrm{F}\mathrm{C}}{(\mathrm{C}\mathrm{F}-\mathrm{C})}$$

$$\mathrm{C}\mathrm{F}=1.25\times(\mathrm{C}+0.25\mathrm{C})$$

BEP = Break-even point, h/year.

FC = Annual fixed cost, ₹/year.

CF = Custom fee, ₹/h.

C = Operating cost, ₹/h.

#### Payback period

The Payback period (PBP) is the duration needed to recover the initial investment by using the cash flows generated from that investment.

$$\mathrm{P}\mathrm{B}\mathrm{P}=\frac{\mathrm{I}\mathrm{C}}{\mathrm{A}\mathrm{N}\mathrm{P}}$$

PBP =Payback period, year.

IC = Initial cost of machine, ₹.

$$\mathrm{A}\mathrm{N}\mathrm{P}=\left(\mathrm{C}\mathrm{F}-\mathrm{C}\right)\times\mathrm{A}\mathrm{U}$$

ANP = Annual net profit.

AU = Annual utility, h/year

$$\mathrm{A}\mathrm{U}=\mathrm{A}\mathrm{A}\times\mathrm{E}\mathrm{C}$$.

AA = Average annual use, h/year.

EC = Effective capacity of machine, ha/h.

### Statistical analysis

The experimental design and statistical evaluation of the data were performed using Response Surface Methodology (RSM) based on a Central Composite Design (CCD). A second-order polynomial regression model was developed for each response variable, namely cell fill (Fc), qualified rate of single cell (Qs), missing cell percent (Cmiss), and multiple cell percent (Cmult), to describe the relationship between the independent variables and the performance parameters.

Analysis of variance (ANOVA) was conducted to assess the significance of the developed regression models and the individual model terms. The effects of linear, interaction, and quadratic components were examined. Model terms were considered statistically significant at *p* < 0.05, while highly significant effects were identified at *p* < 0.01.

The adequacy of the fitted models was further evaluated by comparing the predicted responses obtained from the regression equations with the corresponding results from EDEM simulation and laboratory experiments under optimized operating conditions. The percentage deviation between predicted and observed values was calculated to assess prediction accuracy. The validated regression models were subsequently used for numerical optimization of the operating parameters.

## Results and discussion

### Analysis of angle of repose results

Under the condition of hollow cylinder method, the angle of repose during the physical experiment was observed as 32.04°, 31.97° and 33.94°, with an average of 32.65°. the simulated angle of repose was observed as 30.50°. In comparison to physical measurement, the relative error in simulation was found as 6.58%, which is within reasonable range^40^.

### Effect of input parameters on performance parameters

The simulation of the metering system was performed using EDEM software. The data required (cell fill, qualified rate of single cell, missing cell and multiple cell percent) was obtained through simulation conducted in EDEM. A calibrated EDEM model was utilized to conduct experiments under specific independent parameter settings, and the corresponding results were calculated (Table 7).

**Table 7:** List of input parameters and corresponding responses.

Sl no.	Input parameters			Responses			
	Area	Speed	Hopper fill	F_c_	Q_s_	C_miss_	C_mult_
R1	1325	0.22	75	142	72	2	16
R2	795	0.34	25	62	95	19	2
R3	1060	0.28	50	86	92	6	6
R4	795	0.22	25	72	90	11	3
R5	1060	0.28	100	82	96	5	7
R6	1060	0.28	0	78	89	6	4
R7	1325	0.34	75	116	76	7	11
R8	1060	0.28	50	86	92	7	8
R9	1060	0.28	50	103	88	7	7
R10	795	0.34	25	58	95	19	4
R11	1060	0.28	50	86	92	5	8
R12	530	0.28	50	56	97	22	2
R13	1325	0.22	25	128	68	1	12
R14	1325	0.34	75	121	78	4	14
R15	795	0.34	75	61	98	18	3
R16	1590	0.28	50	164	61	1	16
R17	795	0.22	25	64	91	10	6
R18	1325	0.22	25	130	69	1	14
R19	795	0.22	75	81	96	5	4
R20	1325	0.22	75	138	77	9	14
R21	1325	0.34	25	104	70	8	10
R22	1060	0.28	50	98	90	7	8
R23	1060	0.28	50	94	92	6	8
R24	1060	0.28	50	88	90	5	8
R25	1060	0.16	50	86	86	1	12
R26	1060	0.4	50	75	92	16	8
R27	1325	0.34	25	110	82	4	15
R28	1060	0.28	50	86	91	6	7
R29	795	0.22	75	76	95	11	2
R30	795	0.34	75	71	96	18	3

#### Effect of input parameters on cell fill

**Fig. 9:**
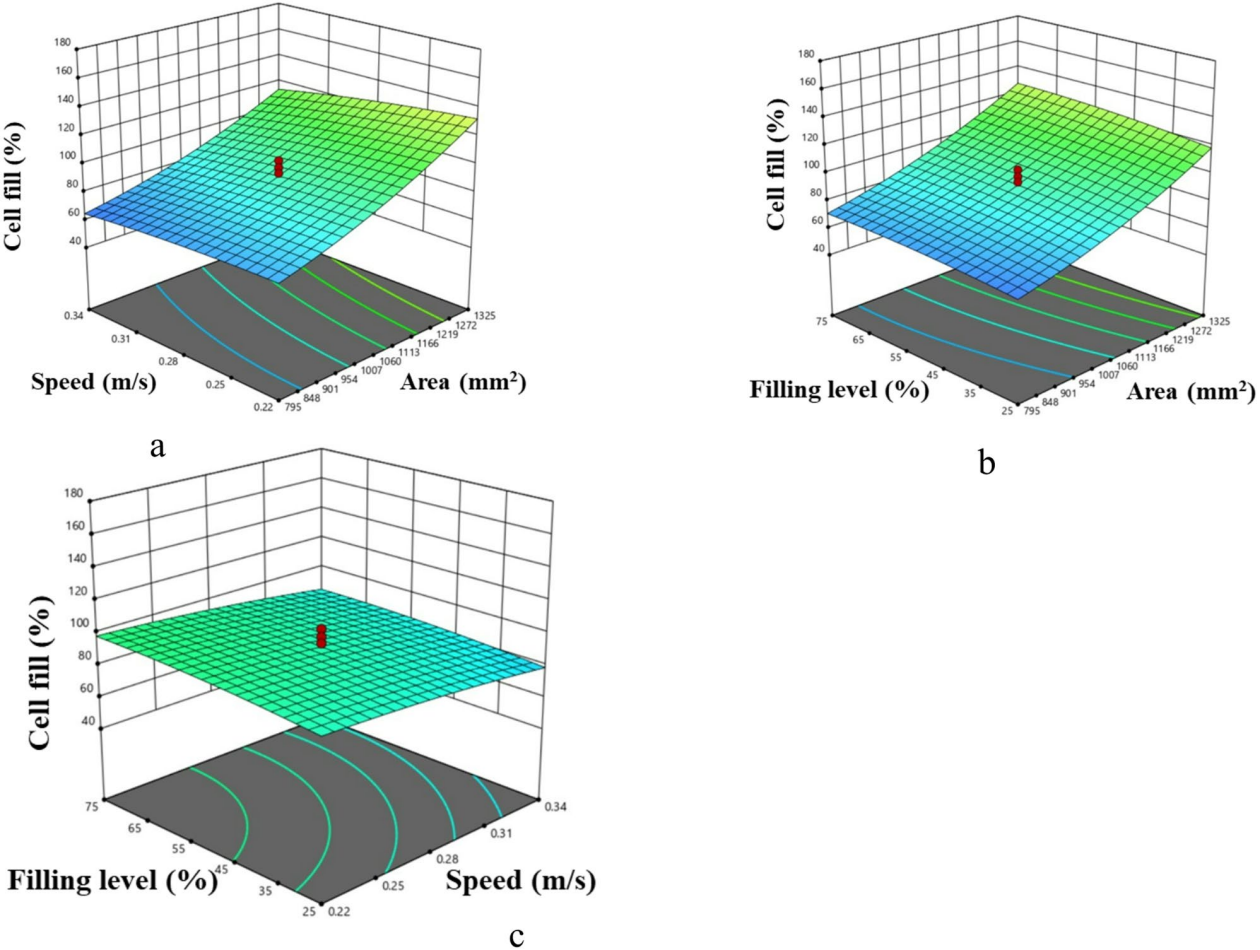
Effect of machine and operating parameters on cell fill.

The total number of USG delivered was calculated by dividing the mean weight of USG to total mass of USG metered as observed by mass flow sensor. Observed values were used to investigate the influence of input parameters on cell fill. Cell area and speed had highly significant effect on cell fill (*P* < 0.01) whereas level of hopper filling affected the cell fill at 5% level (Table 8). An increase in mean cell fill from 88% to 164% was observed as cell area expanded from 1060 mm² to 1590 mm² at a peripheral speed of 0.22 m/s (Fig. 9a). This increase suggests that the larger cell area accommodated a greater number of USGs, resulting in a higher cell fill percentage. Conversely, a 10% reduction in cell fill (from 72% to 62%) occurred for a cell area of 795 mm² when peripheral speed increased from 0.22 to 0.34 m/s (Fig. 9b). This reduction is likely due to the fact that, at lower speeds, cells have more time to fill from the hopper. Increased peripheral speed may induce vibration and reduce pickup time, thus decreasing cell fill rates^17,31,41^. Additionally, cell fill increased from 128% to 142% as hopper fill level rose from 25% to 75% for a metering system with a 1325 mm² cell area at a speed of 0.22 m/s (Fig. 9c). The probable cause might be chance of a cell to be occupied increased with hopper level^42,43^.

**Table 8:** ANOVA for the effect of cell area, peripheral speed, level of hopper filling and gap setting on performance parameters.

Source	F_c_		Q_s_		C_miss_		C_mult_	
	MS	*P*-val	MS	*P*-val	MS	*P*-val	MS	*P*-val
Model	2305.17	< 0.0001*	786.92	< 0.0001*	111.84	< 0.0001*	129.75	< 0.0001*
A	18,150	< 0.0001*	247.04	0.0009*	610.04	< 0.0001*	1027.04	< 0.0001*
B	937.5	0.0002**	234.38	0.0012*	273.38	< 0.0001*	63.38	0.0002**
C	308.17	0.0153**	35.04	0.1596 ^NS**^	0.0417	0.9275^NS**^	5.04	0.2182^NS**^
AB	132.25	0.0978^NS*^	1008.06	< 0.0001*	33.06	0.0173**	3.06	0.3336 ^NS**^
AC	9	0.6553 ^NS**^	3.06	0.6705 ^NS**^	14.06	0.106 ^NS**^	1.56	0.4873 ^NS**^
BC	4	0.7657 ^NS**^	1.56	0.7609 ^NS**^	3.06	0.4388 ^NS**^	0.0625	0.8889 ^NS**^
A²	869.14	0.0002*	4609.31	< 0.0001*	62.25	0.002*	9.43	0.0974 ^NS*^
B²	91.08	0.1649 ^NS**^	1354.56	< 0.0001*	15.38	0.0919 ^NS*^	40.68	0.0017*
C²	104.14	0.1389 ^NS**^	0.1808	0.9175 ^NS**^	0.0022	0.9832 ^NS**^	12.56	0.0585 ^NS*^

*Significant at *p* < 0.01;** Significant at *p* < 0.05;^NS*^, nonsignificant at *P* > 0.05; ^NS**^, nonsignificant at *P* > 0.1; NS is the abbreviation to represent non-significant effects. A = cell area, B = peripheral speed, c = level of hopper filling.

#### Effect of input parameters on qualified rate of single cell of metering system

By visual observation, the number of cells carrying single USG was calculated and by dividing the number of cells passing through the discharge point, qualified rate of single cell was calculated (Table 4). It was observed that, qualified rate of single cell was highly significantly affected by both cell area and peripheral speed of the metering roller (*P* < 0.01) (Table 8). An observed trend indicated that the qualified rate of single cell initially increased with rising cell area and peripheral speed, but subsequently declined beyond certain thresholds^44^.

**Fig. 10:**
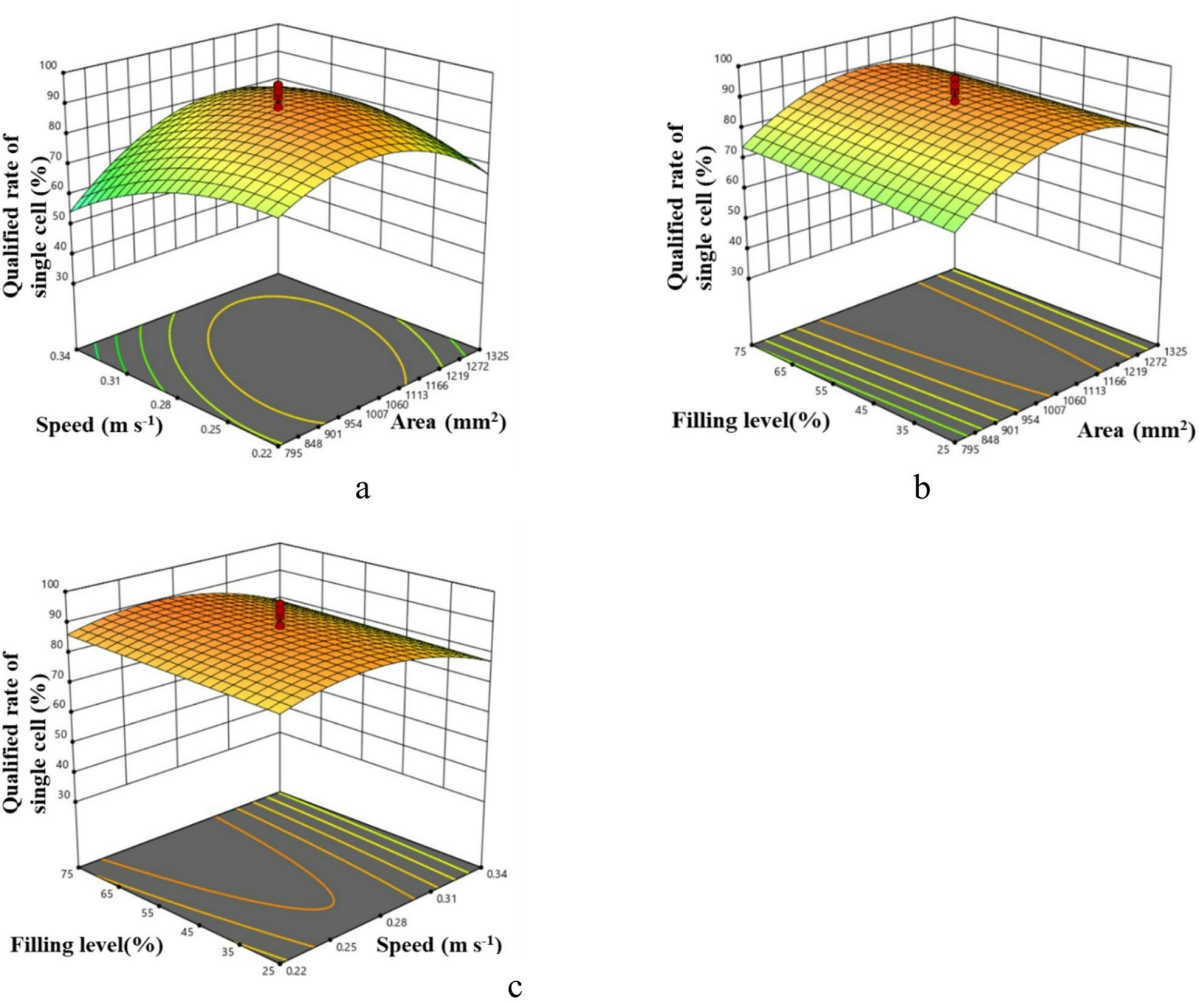
Effect of machine and operating parameters on qualified rate of single cell.

For a metering system with a cell area of 1060 mm², the qualified rate rose from 71% at a speed of 0.16 m/s to a peak mean value of 89% at 0.28 m/s, before decreasing to 57% at 0.4 m/s when the hopper filling level was at 50% (Fig. 10a). This pattern may be explained by lower vibration at reduced speeds, which could promote multiple pickups per cell. At higher speeds, excess USGs picked by the cell were likely expelled, and with further speed increases, even those initially filled in the cell were ejected due to heightened vibrations, resulting in a lower qualified rate. Additionally, as cell area increased from 530 mm² to 1060 mm², the qualified rate of single cell rose from 36% to 89%, though it declined when cell area was further expanded to 1590 mm² (Fig. 10b). This outcome suggests that with smaller cell areas, the probability of USG pickup was lower, but initially improved as cell area grew. However, with further area increase, multiple USGs were more likely picked, thereby reducing the qualified rate of single cell. Hopper fill level did not show any significant impact on the qualified rate of single cell (Fig. 10c)^44^.

#### Effect of input parameters on missing cell percent of metering system

**Fig. 11:**
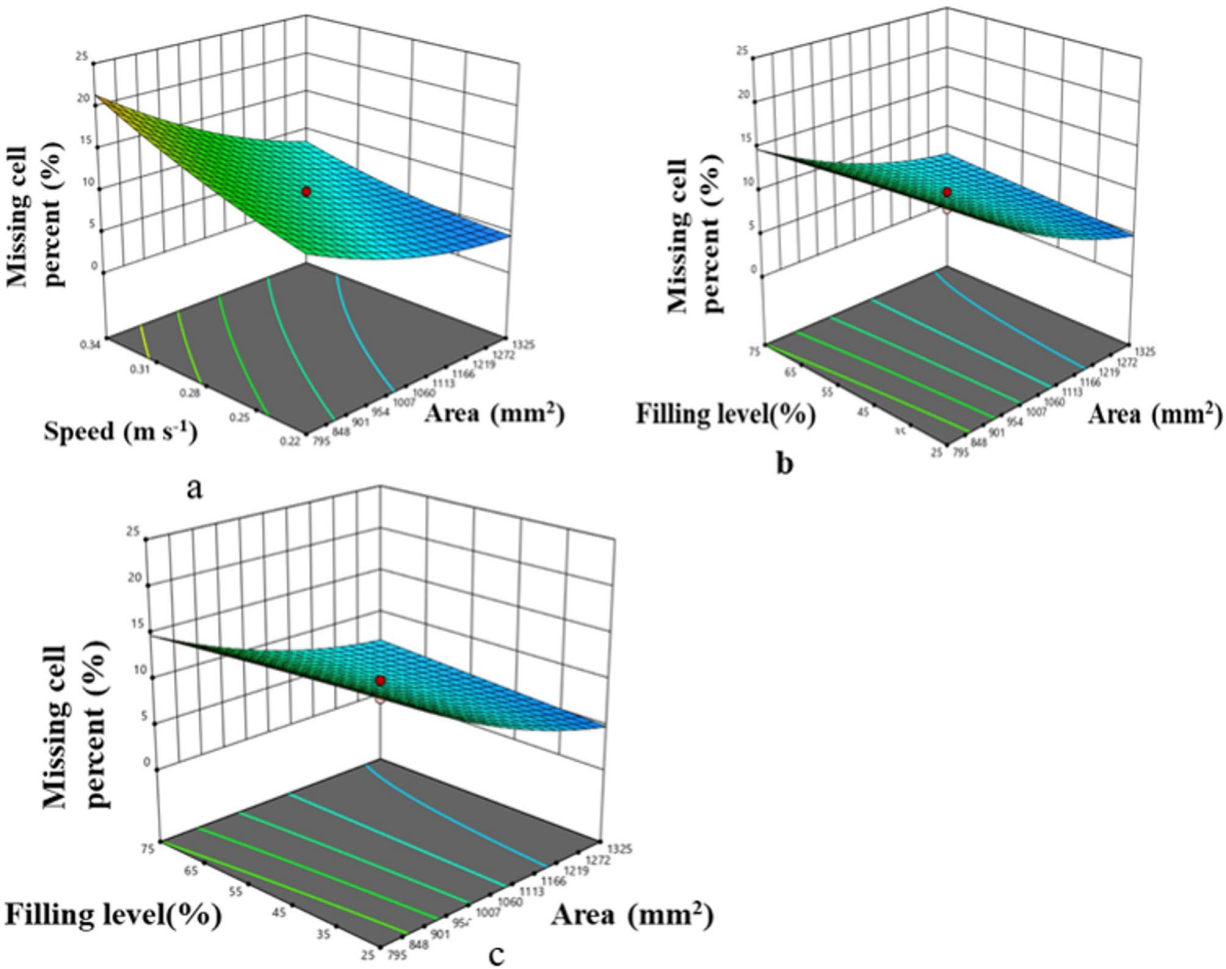
Effect of machine and operating parameters on missing cell percent.

Number of filled cells and missing cells of the metering system over a run of 50 revolution was observed by visual observations. Missing cell percent was calculated by using the Eq. 19. An observed trend indicated that the percentage of missing cells decreased as cell area increased. Specifically, expanding the cell area from 530 mm² to 1060 mm² reduced the missing cell percentage from 18% to 4% at a peripheral speed of 0.28 m/s and a hopper filling level of 50% (Fig. 11a). This decrease can likely be attributed to an increased probability of USG pickup by the metering roller with larger cell areas, consistent with findings from previous studies on pneumatic seed metering systems^45^. In contrast, an increase in peripheral speed was associated with a rise in the percentage of missing cells. For a metering system with a cell area of 1060 mm², the missing cell percentage increased from 2% to 12% as speed rose from 0.16 m/s to 0.4 m/s (Fig. 11b). This increase in missing cell percentage may be due to reduced time for the metering system to collect USGs as peripheral speed rises^45,46^. The level of hopper filling did not have a consistent effect on the percentage of missing cells, though missing cell percentage increased with higher peripheral speeds (Fig. 11c).

#### Effect of input parameters on multiple cell percent

**Fig. 12:**
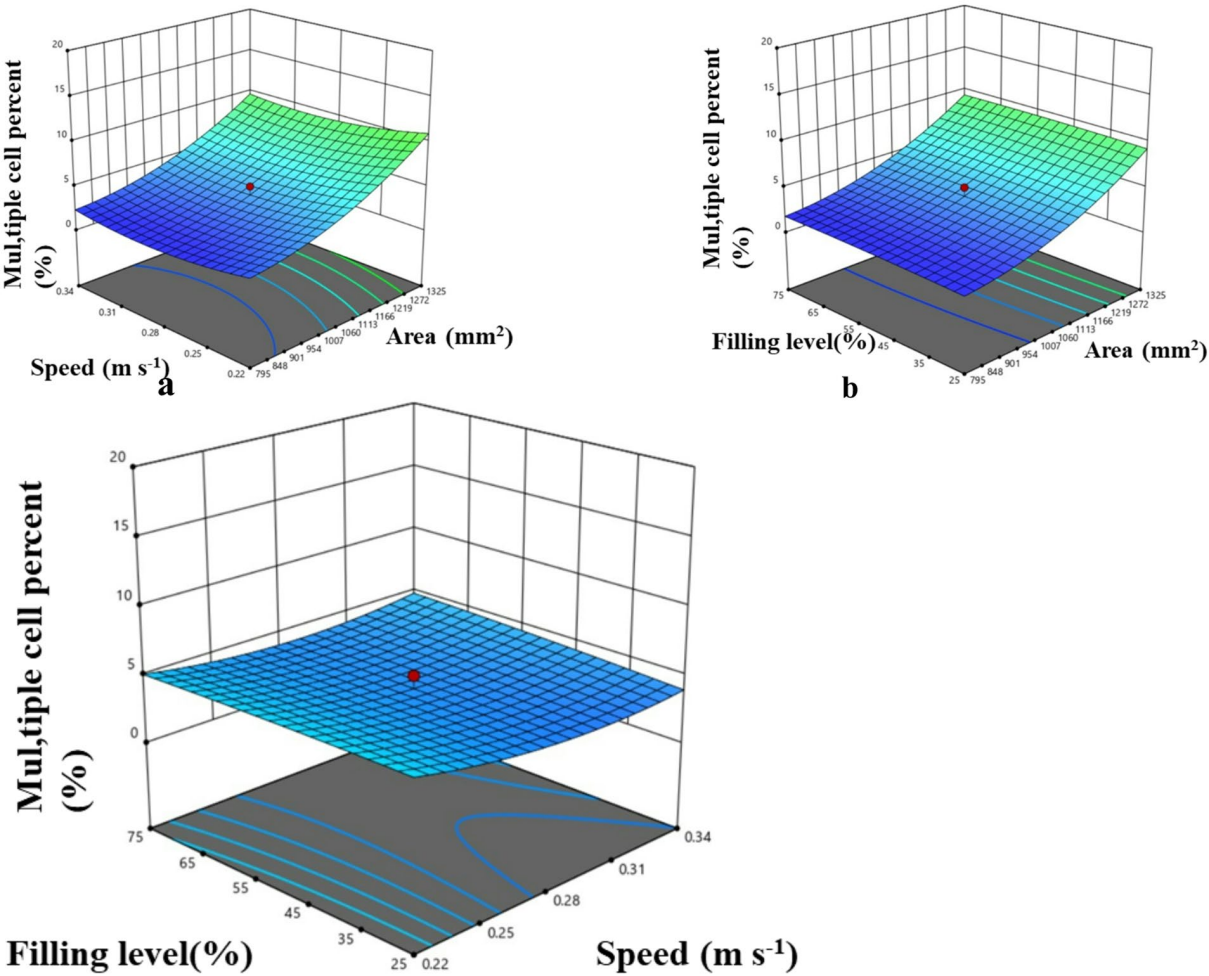
Effect of machine and operating parameters on multiple cell percent.

By visual inspection the number of cells that picked multiple USGs were recorded and multiple cell percent was calculated by using the formula given in Eq. 20. It was inferred that cell area and speed had highly significant effect on multiple cell percent (*P* < 0.01) (Table 8). An increase in cell size was associated with a rise in the percentage of multiple cells. Specifically, expanding the cell size from 530 mm² to 1590 mm² resulted in an increase in multiple cell percentage from 2% to 18% at a peripheral speed of 0.28 m/s and a hopper filling level of 50% (Fig. 12a). This effect likely occurs because larger cell areas increase the likelihood of picking multiple USGs, thereby raising the multiple cell percentage.

^45,47^. When the peripheral speed of the metering system was increased at a constant hopper fill level, the multiple cell percentage initially decreased before rising again. For a cell area of 1060 mm², the multiple cell percentage dropped from 9% to 4% as speed increased from 0.16 m/s to 0.28 m/s, but then rose to 6% at a speed of 0.4 m/s with 50% hopper filling (Fig. 12b, c). As peripheral speed increased, the multiple cell percentage initially declined. At higher speeds, increased vibration of the metering roller and reduced picking time may cause USGs to either be ejected from or not be collected by the cell, leading to a lower multiple cell percentage^17,31^.

### Optimization of input variables

The operating parameters of the metering system were optimized with the objective of achieving maximum cell fill (target: 100%), maximizing the qualified rate of single cell, and minimizing both missing cell percent and multiple cell percent. Since multiple responses were involved, a numerical multi-response optimization approach was employed within the RSM framework. A desirability function methodology was used to simultaneously optimize all response variables. For cell fill and qualified rate of single cell, the goal was set to maximize the response, whereas for missing cell percent and multiple cell percent, the objective was set to minimize the response. The experimental lower and upper bounds of each response were defined based on the observed data. All responses were assigned equal importance during the optimization process, as each performance indicator contributes directly to the overall quality of fertilizer placement. No preferential weighting was introduced to avoid bias toward a specific performance parameter.

The optimal combination of cell area, peripheral speed, and hopper filling level was determined as 1088 mm², 0.24 m s⁻¹, and 75%, respectively. Under these conditions, the predicted values obtained from the RSM model were 100% for cell fill, 89.18% for qualified rate of single cell, 3.01% for missing cell percent, and 4.84% for multiple cell percent (Table 9).

**Table 9:** Optimum operational parameters and performance parameters predicted by RSM.

Optimum operational parameters			Predicted performance parameters			
Area (mm^2^)	Speed (m/s)	Level (%)	F_c_ (%)	Q_s_ (%)	C_miss_ (%)	C_mult_ (%)
1088	0.24	75	100	89.18	3.01	4.84

### Validation results of optimum parameters through laboratory evaluation and EDEM simulation

The optimized conditions determined through RSM were utilized as input parameters for both laboratory experiments and EDEM simulations. For the laboratory experiment, a metering system with a cell area of 1088 mm² was fabricated using 3D printing. The speed of the metering roller was precisely controlled via a stepper motor, which was programmed using Arduino. Under optimized conditions, the laboratory experiment yielded observed values for cell fill, single cell qualified rate, missing cell percentage, and multiple cell percentage of 97%, 91%, 3.2%, and 4.5%, respectively (Table 10).

Similarly, the optimized input parameters derived from RSM were applied in the EDEM simulation. The simulation results indicated values of 97% for cell fill, 93% for the single cell qualified rate, 2.9% for missing cell percentage, and 4.7% for multiple cell percentage (Table 10). The deviations between the predicted data from RSM and the observed data from EDEM simulations were calculated, demonstrating the accuracy and effectiveness of both RSM and EDEM in predicting the performance of the metering system.

**Table 10:** Predicted and observed performance parameters at optimized operational conditions.

Performance parameters	Predicted value		Observed value	Deviation (% error)	
	RSM	EDEM	Lab evaluation	RSM	EDEM
Cell fill (%)	100	97	97	3	3
Qualified rate of single cell (%)	89.18	93	91	2	2.1
Missing cell (%)	3.01	2.9	3.2	5.9	9.3
Multiple cell (%)	4.84	4.7	4.5	7.55	4.44

### Performance of developed applicator system under laboratory conditions

**Fig. 13:**
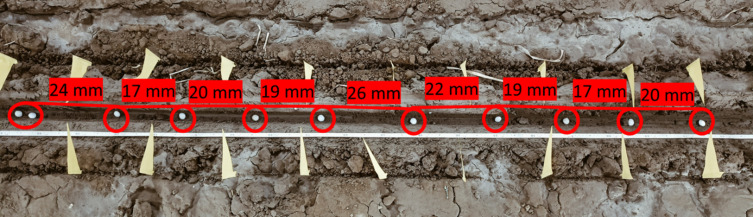
USG distribution in the furrow.

The applicator system was operated under optimized conditions, and the distance between two consecutive USGs was measured along a run length of 10 m to evaluate the system performance (Fig. 13). The applicator was run for 10 repetitions, and mean value of missing index, multiple index, and quality of feed index were observed as 4.61%, 4.23% and 91.16% respectively and application uniformity quality of USG was classified as good (Table 11)^16^.

**Table 11:** Quality class of metering system observed under laboratory study.

Parameters	Observed value	Classification
Missing index	3.2	Good
Multiple index	4.5	Good
Quality of feed index	92.3	Good

### Cost of economics of developed USG applicator

The cost of operation the retrofitted prototype USG applicator was calculated following the guidelines outlined in Sect. 3.12 of IS: 9164 − 1979: Guide for estimating the cost of farm machinery operation (Appendix XVIII). The analysis led to the determination of the final cost of the prototype, the total cost of operation per hour, and the cost of operation per hectare. Moreover, the break-even point (BEP) and payback period (PBP) of the developed applicator were estimated as 64 h/year and 1.5 year respectively (Table 12).

**Table 12:** Cost economics of developed prototype USG applicator.

Prototype machine	Details
Cost of prototype, ₹	19,420
Hourly cost of operation with transplanter, ₹	509
Break-even point	64 h/year
Payback period, year	1.5

## Conclusions

To reduce human drudgery involving application of USG, a mechanical applicator is need of the hour. Metering system is an important component for precision application of USG. Therefore, the study was conducted to optimize the mechanical and operational parameters of the metering system. The operation of the metering was simulated using EDEM software, while the optimization of input parameters was conducted through the Response Surface Methodology (RSM). The independent variables in this study were cell area, roller peripheral speed, and hopper fill level. A quadratic RSM model was developed to fit the response variables, which included cell fill, qualified rate of single cell, missing cell percent and multiple cell percent. The optimization of these independent variables aimed to achieve a target cell fill as close to 100% as possible, maximize the qualified single-cell rate, and minimize the percentage of both missing and multiple cells. Finally, the soil bin experiment was carried out with the optimized parameters. Research findings from the study has been summarized below.

1. It was observed that RSM and EDEM model can accurately predict the performance of metering mechanism.
2. Cell area had significant effect on all responses. Increasing cell area increase in cell fill, multiple cell percent was observed whereas qualified rate of single cell was increased first and then decreased and missing cell percent decreased.
3. Peripheral speed of metering system had significant effect on all the responses. Increasing speed cell fill, multiple index and coefficient of hill distribution decreased.
4. Level of hopper filling had significant effect only on cell fill and did not have significant effect on other responses. Increasing hopper filling increased cell fill.
5. Optimized set of operating parameters were found as 1088 mm^2^ cell area, 0.24 m s^−1^ metering roller peripheral speed and 75% fill of hopper. At these optimized settings, the cell fill, qualified rate of single cell, missing cell and multiple cell were found to be 97%, 91%, 3.2% and 4.5%.
6. Missing index, multiple index and quality of feed index at the optimized set of operating parameters of metering system were observed as 3.2%, 4.5% and 92.3%, respectively and the performance was categorized as good.

## Data Availability

The datasets used and/or analysed during the current study are available from the corresponding author on reasonable request.
